# Occupational health risk assessment of workplace solvents and noise in the electronics industry using three comprehensive risk assessment models

**DOI:** 10.3389/fpubh.2023.1063488

**Published:** 2023-03-17

**Authors:** Qifan Huang, Shibiao Su, Xiaoshun Zhang, Xiang Li, Jiawei Zhu, Tianjian Wang, Cuiju Wen

**Affiliations:** ^1^Institute of Occupational Health Assessment, Guangdong Province Hospital for Occupational Disease Prevention and Treatment, Guangzhou, China; ^2^School of Public Health, Sun Yat-sen University, Guangzhou, China; ^3^School of Public Health, Southern Medical University, Guangzhou, China; ^4^Department of Management of Research and Education, Guangdong Province Hospital for Occupational Disease Prevention and Treatment, Guangzhou, China

**Keywords:** electronics industry, occupational health, risk assessment, comprehensive risk, solvent, noise

## Abstract

**Background:**

Occupational hazards such as solvents and noise in the electronics industry are serious. Although various occupational health risk assessment models have been applied in the electronics industry, they have only been used to assess the risks of individual job positions. Few existing studies have focused on the total risk level of critical risk factors in enterprises.

**Methods:**

Ten electronics enterprises were selected for this study. Information, air samples and physical factor measurements were collected from the selected enterprises through on-site investigation, and then the data were collated and samples were tested according to the requirements of Chinese standards. The Occupational Health Risk Classification and Assessment Model (referred to as the Classification Model), the Occupational Health Risk Grading and Assessment Model (referred to as the Grading Model), and the Occupational Disease Hazard Evaluation Model were used to assess the risks of the enterprises. The correlations and differences between the three models were analyzed, and the results of the models were validated by the average risk level of all of the hazard factors.

**Results:**

Hazards with concentrations exceeding the Chinese occupational exposure limits (OELs) were methylene chloride, 1,2-dichloroethane, and noise. The exposure time of workers ranged from 1 to 11 h per day and the frequency of exposure ranged from 5 to 6 times per week. The risk ratios (RRs) of the Classification Model, the Grading Model and the Occupational Disease Hazard Evaluation Model were 0.70 ± 0.10, 0.34 ± 0.13, and 0.65 ± 0.21, respectively. The RRs for the three risk assessment models were statistically different (*P* < 0.001), and there were no correlations between them (*P* > 0.05). The average risk level of all of the hazard factors was 0.38 ± 0.18, which did not differ from the RRs of the Grading Model (*P* > 0.05).

**Conclusions:**

The hazards of organic solvents and noise in the electronics industry are not negligible. The Grading Model offers a good reflection of the actual risk level of the electronics industry and has strong practicability.

## Background

The electronics industry is a strategic emerging industry in China, and its production process is characterized by rapid renewal and complex intermediate products. The Chinese electronics industry has a wide range of occupational hazards, a large number of employees, and a high risk of occupational disease. There exists a coexistence of outdated and advanced production processes (1). Wen et al. (2) analyzed the disease spectrum of new occupational diseases in Guangdong Province from 2006 to 2010, and the number of new cases in the electronics industry ranked third among all industries. Tian et al. (3) measured the noise intensity exposure of job positions in electronics enterprises and combined it with the results of workers' health examinations for a comprehensive analysis. It was found that the noise exceedance rate was relatively high, and nearly half of the workers had abnormal pure-tone audiometric results, suggesting that noise may be a significant hazard factor in this industry. Yang et al. (4) explored the exposure to organic solvents and found a variety of organic solvents in the electronics industry, such as *n*-hexane and benzene. In summation, workers in the electronics industry are facing high occupational health risks, particularly exposure to noise and organic solvents, and supervision and management should be strengthened by regulations and employees.

Occupational health risk assessment is considered an essential tool for maintaining the health of workers (5). As a result, many countries and organizations have developed various occupational health risk assessment models, including the Singapore model (6), the US EPA quantitative risk assessment model (7), the ICMM model (8), the Romanian risk assessment model (9), and the COSHH essential model (10). Previous studies (11–14) have explored whether occupational health risk assessment models can be applied in the electronics industry, to provide scientific guidance for enterprises to accurately identify high-risk positions and take appropriate control measures. Xu et al. (15) and Tian et al. (16) used quantitative or qualitative–quantitative methods to explore the consistency, relevance, and other indicators of the assessment results of six models commonly used for risk assessment in the electronics industry and established a more comprehensive framework for model comparison.

However, traditional models evaluate the risk levels of specific job positions, and although the results may be highly accurate, they are not always useful for helping occupational health regulators decide which enterprises require intervention. Therefore, some studies have developed comprehensive risk assessment methods for evaluating the overall occupational health risks of enterprises. A comprehensive risk assessment method was mentioned in the Romanian risk assessment model, where the comprehensive risk level in the workplace was calculated from weighted average of the identified risk factors. Li et al. (17) proposed a new method of occupational health risk assessment based on Set Pair Analysis, which could assess the comprehensive risk of welding workshops. Jahangiri et al. (18) used a comprehensive occupational health risk assessment model to prioritize occupational health hazards in petrochemical companies and to determine resource allocation and required control measures. Ji et al. (19) in New Zealand revised the conventional risk assessment approach to a comprehensive risk assessment method that considered both safety accidents and chronic health issues, providing a way to incorporate long-term health outcomes into occupational health risk accessment. The purpose of this study was to explore the application of three Chinese comprehensive risk assessment models to the electronics industry in China based on the hazard characteristics of the electronics industry, organic solvents and noise, and to quantitatively compare the difference and correlation of their assessment results to provide new ideas for implementing efficient occupational health supervision.

## Materials and methods

### Description of study subjects

To obtain a large sample size and fully reflect the characteristics of the production process in the electronics industry, 10 electronics enterprises in Shenzhen, Guangdong Province, China, were selected for the study, with a labor quota distribution of 350–1,000 employees and a complex range of major products, including electronic sports watch-related accessories, relays, computers, printers, LCD monitors, printing consumables, hard disk drive components, connectors, printed circuit boards, inductors, conductive silicone, and inverters.

### Site survey and on-site testing

In this study, a uniform questionnaire was used to investigate the basic information, production process, production system of each position, daily exposure time, weekly exposure days, and occupational health management of each enterprise. Air sampling for chemical poisons was performed according to the Chinese sampling standard described in “*The sampling specification for hazardous substances monitoring in workplace air* (GBZ 159-2004)” (20). Laboratory testing of these chemicals was based on “*The determination of toxic substances in the workplace* (GBZ/T 160-2004)” (21) series of standards. The 8-h time-weighted average concentration (C-TWA) of chemical toxicants were tested and compared with the permissible concentration-time weighted average (PC-TWA) in the Chinese standard “*Occupational exposure limits for hazardous agents in the workplace Part 1: Chemical hazardous agents* (GBZ 2.1-2019)” (22). Field measurements of noise were conducted according to the standard “*The physical factor measurement in the workplace* (GBZ/T 189.8-2007)” (23). If the work shift was 5 days per week, the noise exposure level was defined as the equivalent continuous A-weighted sound pressure level normalized to a nominal 8 h working day, *L*_EX,8h_. If the work shift was not 5 days per week, the equivalent continuous A-weighted sound pressure level normalized to a nominal 40 h working week, *L*_EX,W_ was used to reflect the noise exposure level.

### Risk assessment models

In this study, three models were used to assess the comprehensive risks of 10 electronics enterprises, including two newly developed comprehensive risk assessment models—the Occupational Health Risk Classification and Assessment Model (referred to as the Classification Model) and the Occupational Health Risk Grading and Assessment Model (referred to as the Grading Model)—and the Occupational Disease Hazard Evaluation Model used in China. The risk assessments of the three models were completed by professional occupational health institutions or relevant departments of the enterprises.

1. The Classification Model. This model was developed by the National Administration of Disease Prevention and Control, PRC, and the National Health Commission, PRC. According to the requirements of the Chinese guideline, “*Guidelines for Occupational Health Risk Classification and Grading Assessment of Employers*.” the comprehensive risks of the 10 selected enterprises were devided into levels A, B, and C from high to low risk. The detailed principles are shown in Table 1.
2. The Grading Model. This model was developed by Chinese scholars with reference to various occupational health risk assessment models such as the Singapore model, the ICMM model, and the Romanian model, and adjusted based on the management situation of enterprises. The Grading Model was applied in this study as follows. First, the risk level of each occupational disease hazard was determined by the hazard rating (HR) and exposure rating (ER). HRs and ERs of chemical hazard factors could be identified according to the Singapore model. The calculation of ER depended on the ratio of the weekly exposure *E* to the occupational exposure limit (OEL). *E* was calculated using the equation: $E=\frac{F\times D\times M}{W}$, where *F* is the frequency of exposure per week, *M* is the magnitude of exposure, *W* is the average working hours per week, *D* is the average duration of each exposure. Depending on the severity of the health effects of the hazards (in the order of minor health effects, reversible health effects, permanent irreversible health effects, significant and severe health effects, and death), the HRs of physical factors were classified into five classes according to the method described in the ICMM model. As shown in Table 2, the ER of noise was graded according to the A-weighted equivalent sound pressure level (*L*_Aeq_). *R*_*i*_ was interpreted as the risk level of different hazards in the position. Due to the complexity of the types of hazards present in the workplace, *R*_*i*_ had multiple values. And *R*_*i*_ was calculated by the equation: $R_{i}=\sqrt{HR\times ER}$. In addition, according to the Romanian risk assessment model, the comprehensive risk level *R*_*O*_ was calculated by a weighted average of *R*_*i*_ for each position through the equation $R_{o}=\frac{\Sigma_{i=1}^{n}R_{i}\times r_{i}}{\Sigma_{i=1}^{n}r_{i}}$. Finally, the 12 major items—such as the management measures for occupational disease prevention and control, declaration of occupational disease hazards, “three simultaneous” of occupational disease protection facilities in construction projects, and occupational health conditions in the workplace—were checked and assigned scores, using the self-inspection form for the implementation of occupational disease prevention and control responsibilities of the enterprises in the appendix of the Chinese guideline “*Occupational Health Risk Classification and Assessment Guide for Employers*.” The ratio of the actual score to the total score was the standardized score. The standardized score divided the Occupational Health Management Index (MI) into four levels: A (90–100 points), B (80–89 points), C (70–79 points), and D (<70 points). Referring to the matrix method of the COSHH essential model, the matrix shown in Table 3 was used to determine the adjusted comprehensive risk *R*_*O*_' of the enterprises.
3. The Occupational Disease Hazard Evaluation Model (24). This model was established by combining the Occupational Hazards Risk Assessment Index Method (25) with the occupational health management level of an enterprise. According to this model, the comprehensive risk level of the enterprise depends on the two key indicators, the occupational hazard risk index grade and management quality. Therefore, in this study, the occupational hazard risk index was calculated using the formula: risk index = 2^health effect level^ × 2^exposure ratio^ × operating condition level and then divided into five levels according to the risk index, no hazard (risk index ≤ 6), mild hazard (6 < risk index ≤ 11), moderate hazard (11 < risk index ≤ 23), high hazard (23 < risk index ≤ 80), and extreme hazard (risk index > 80). By calculating the weights to grade the occupational health management of enterprises, the management quality was divided into five categories: fail, pass, average, good, and excellent, using 0.6, 0.7, 0.8, and 0.9 as the boundary. The risk index grade and the management quality grade were used to construct a matrix to determine the comprehensive risk level, as shown in Table 4.

**Table 1:** The detailed principle of the classification model.

**Category**	**Principle**
**A**	Enterprises belong to specific industries such as mining, manufacturing, electricity, heat, gas and water production and supply, etc. Industry classification refers to the National Economic Classification and Codes (Chinese standard: GB/T 4754-2017).
	1. The presence of high-risk chemicals such as hydrogen cyanide, n-hexane, aniline in the workplace, the concentration of which reaches or exceeds 50% OEL.
	2. The presence of benzene, 1,2-dichloroethane, trichloroethylene, etc. in the workplace in concentrations of 10% OEL or more.
	3. The workplace has chemical substances or productive dust (free silica ≥10%) in excess of the OEL.
	4. Workplace with nuclear facilities, irradiation processing equipment, radiation therapy equipment, industrial flaw detection machines, oilfield logging equipment.
	5. Newly diagnosed occupational diseases within the last 2 years.
	6. Enterprises included in the scope of key management by the health administration.
	7. Enterprises that meet one of the above conditions are included in Category A.
**B**	The presence of highly hazardous chemicals in the workplace, such as hydrogen cyanide, n-hexane, aniline, etc. in concentrations below 50% OEL.
	1. The presence of benzene, 1,2-dichloroethane, trichloroethylene, etc. in the workplace in concentrations below 10% OEL.
	2. The presence of chemical substances or productive dust (free silica ≥ 10%) in the workplace.
	3. The presence of radioactive occupational disease hazards in the workplace.
	4. Enterprises that meet one of the above conditions are included in Category B.
**C**	Occupational hazards exist in the workplace, but the enterprise is not classified as Category A or Category B.

**Table 2:** The rank of noise exposure.

**Definition (dB(A))**	**Exposure rank (ER)**
L_Aeq_ < 75	1
75 ≤ L_Aeq_ < 80	2
80 ≤ L_Aeq_ < 85	3
85 ≥ L_Aeq_ < 90	4
L_Aeq_ ≥ 90	5

**Table 3:** Comprehensive risk matrix for the grading model.

**The comprehensive risk level R_O_**	**The occupational health management index (MI)**			
	**Grade D**	**Grade C**	**Grade B**	**Grade A**
1	2	1	1	1
2	2	2	1	1
3	3	3	2	2
4	4	4	3	3
5	4	4	4	4

**Table 4:** The comprehensive risk matrix for the occupational disease hazard evaluation model.

**Management quality (MQ)**	**Occupational hazard risk index grade**				
	**No hazards**	**Mild hazards**	**Moderate hazard**	**Highly hazard**	**Extreme hazard**
Excellent	A	A	B	C	C
Good	A	B	B	C	D
General	B	B	C	D	D
Passing	B	B	C	D	D
Failure	B	C	D	D	D

### Comparison between different assessment models

Due to the inconsistent presentation of risk assessment results obtained from different models, the risk levels of the three models were appropriately converted in this study to facilitate comparison. First, categories A, B, and C in the results of the Classification Model were converted to levels 3, 2, and 1. Next, levels A, B, C, and D in the results of the Occupational Disease Hazard Evaluation Model were converted to levels 1, 2, 3, and 4. Then, the risk level of each model was standardized using the risk ratio (RR) definerd by Zhang et al. (26). RR was the ratio between the risk level of a given risk factor obtained from each model and the total risk level of the model, and it was obtained from the formula, *RR* = Actual risk level/Total risk level. As an example, if a particular model's risk level was divided into five levels and level 3 was determined by utilizing the model to evaluate a risk factor, the RR would be equal to 0.6 (3/5).

### Accuracy validation of model results

In this study, an attempt was made to validate the results of three comprehensive risk assessment models using the average risk level of all of the hazard factors for 10 enterprises. Organic solvents were assessed using the Singapore semi-quantitative model, and noise was assessed using an ICMM matrix model. The selection of the above models was based on relevant studies (27) and discussions with experts.

### Statistical analysis

SPSS 23.0 software (IBM, Armonk, NY, USA) was used for statistical analysis. Spearman rank correlation analysis was used to compare the correlations of RRs among the three models. Wilcoxon signed rank sum test was performed on *R*_*O*_ (the Grading Model) and the risk indices (the Occupational Disease Hazard Evaluation Model). Meanwhile, Wilcoxon signed rank sum test was performed on the standardized occupational health management levels between the Grading Model and the Occupational Disease Hazard Evaluation Model. One-way analysis of variance (ANOVA) was used to analyze the RRs in the three models and the mean RR levels of all of the risk factors. The LSD comparison method was used when the variances were equal, and the Dunnett T3 comparison method was used when there was heterogeneity in the variances.

## Results

### On-site occupational survey

Table 5 describes the basic information of the 10 enterprises. The number of exposed workers ranged from 308 to 1,929. Three enterprises had single-shift work, five had two-shift work, and the rest had both shift patterns. Workers in these 10 enterprises were exposed to occupational hazards from 1 to 11 h per day, and the frequency of exposure was 5–6 times per week.

**Table 5:** Basic information of 10 electronics enterprises.

**Enterprise**	**Number of exposed workers**	**Shift system**	**Work hour per day**	**Work day per week**	**Automation level**
A	200	Single shift, two shifts	8	5	Semi-automation
B	1,929	Two shifts	11	5	Semi-automation
C	550	Two shifts	10	6	Semi-automation
D	615	Two shifts	10	6	Semi-automation
E	678	Two shifts	10.5	6	Semi-automation
F	498	Single shift, two shifts	10	6	Semi-automation
G	1,100	Two shifts	10.5	5	Semi-automation
H	400	Single shift	8	5	Semi-automation
I	308	Single shift	8	5	Semi-automation
J	500	Single shift	8	6	Semi-automation

The exposure levels of organic solvents and noise in the 10 enterprises are shown in Table 6. Hazards with concentrations exceeding OEL were methylene chloride, 1,2-dichloroethane, and noise. The one hazard at a concentration above 50% OEL but below OEL was isopropyl alcohol. Hazards with concentrations above 10% OEL but below 50% OEL were methanol, tetrahydrofuran, methanol, isopropanol, ethanolamine, n-hexane, methanol, formaldehyde, xylene, and toluene. The concentrations of other hazards were <10% OEL.

**Table 6:** The result of hazard exposure level in 10 enterprises.

**Enterprise**	**Hazards exposure**			
	<**10%OEL**	**10%OEL-50%OEL**	>**50%OEL**	>**OEL**
A	Benzene, ethylbenzene, hexane, methanol, ethanol, acetone, dichloromethane	–	–	–
B	Benzene, methylbenzene, xylene, ethylbenzene, methanol, butanone, ethyl acetate, butyl acetate, N,N-dimethylacetamide	–	Isopropanol	Noise
C	Isopropanol, methylbenzene, methanol, ethanol	–	–	–
D	Acetone, butanone, methyl benzene	Methanol, tetrahydrofuran	–	Dichloromethane, 1,2-dichloroethane, noise
E	Ethanol	Methanol, isopropanol, ethanolamine	–	–
F	Methylbenzene, cyanide, hydrogen cyanide, sulfuric acid, hydrochloric acid	–	–	Noise
G	Ethanol, methyl benzene, xylene, methyl acetate, ethyl acetate	Hexane, methanol, formaldehyde	–	Noise
H	Benzene, methylbenzene, ethylbenzene, hexane, cyclohexane, methanol, isopropanol, butanol, trichloroethylene	Xylene	–	Noise
I	Benzene, xylene, hexane, cyclohexanone, methanol, ethyl acetate, butyl acetate, isoflurone	Methyl benzene	–	–
J	Benzene, xylene, ethyl benzene, hexane, cyclohexane, methanol, acetone, butanone, dichloromethane, ethyl acetate, butyl acetate, isoflurone, trichloroethylene, tetrachloroethylene	Methyl benzene	–	Noise

As shown in Table 7, six enterprises were found to have hazard factors exceeding the OELs. Among these enterprises, one chemical factor, methylene chloride, exceeded the OEL, with an 8 h time-weighted average concentration (C-TWA) of 331.84 mg/m^3^. The noise exposure intensity of different job positions ranged from 80.8 to 91.9 dB(A). In addition, only two enterprises were found to be fully equipped with health engineering protection and personal protective equipment, accounting for 20% of the total, which indicated that the levels of occupational health management of the enterprises were deficient.

**Table 7:** On-site occupational health survey of 10 electronics enterprises.

**Item**	***N***	**Number of enterprise passed**	**Passing rate**
Occupational hazards	10	4	40.00%
Engineering protections	10	2	20.00%
Personal protective equipment	10	2	20.00%
Emergency rescue facilities	10	2	20.00%
Occupational health management	10	2	20.00%

### Risk assessment results

The risk assessment results of the three risk assessment models are listed in Table 8. The Classification Model classified the 10 enterprises into level 2 (category B) and level 3 (category A); one enterprise belonged to category A and the remaining nine belonged to category B. The Grading Model classified the 10 electronic enterprises into level 1, level 2, and level 3; five enterprises were classified as level 1, four were classified as level 2, and one was classified as level 3. The Occupational Disease Hazard Evaluation Model classified the 10 electronic enterprises into level 1, level 2, level 3, and level 4; one enterprise was in level 1, three enterprises were in level 2, five enterprises were in level 3, and one enterprise was in level 4.

**Table 8:** Results of three occupational risk assessment models.

**Enterprise**	**The classification model**		**The grading model**		**The occupational disease hazard evaluation model**	
	**The result of risk assessment**	**RR**	**The result of risk assessment**	**RR**	**The result of risk assessment**	**RR**
A	2	0.67	2	0.40	4	1.00
B	2	0.67	1	0.20	3	0.75
C	2	0.67	2	0.40	2	0.50
D	3	1.00	2	0.40	2	0.50
E	2	0.67	1	0.20	1	0.25
F	2	0.67	3	0.60	2	0.50
G	2	0.67	2	0.40	3	0.75
H	2	0.67	2	0.40	3	0.75
I	2	0.67	1	0.20	3	0.75
J	2	0.67	1	0.20	3	0.75

### Correlation analysis of the three models

The results of Spearman correlation analysis of the three model presented in Table 9, indicated that there were no correlations between the risk assessment results of all three models, and the difference was not statistically significant (correlation coefficients 0.192, −0.314, and −0.109, respectively; *P* > 0.05).

**Table 9:** Correlation analysis of RRs for three models.

**Variants**	**The classification model**	**The grading model**	**The occupational disease hazard evaluation model**
The classification model^a^	1.000		
The grading model^a^	0.192	1.000	
The occupational disease hazard evaluation model^a^	−0.314	−0.109	1.000

a Compare with each other, if the symbols (like “a”) are same, *P* > 0.05.

### Quantitative differences in the risk ratios between the different models

**Figure 1:**
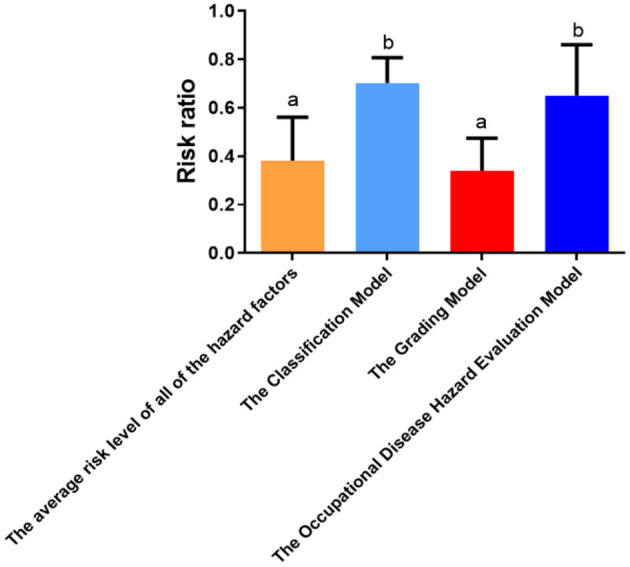
Quantitative differences in the risk ratios between the models. Compare with each other, if the symbols (like “a”) are same, *P* > 0.05.

As shown in Figure 1, the RR for the Classification Model was 0.70 ± 0.10, the RR for the Grading Model was 0.34 ± 0.13, and the RR for the Occupational Disease Hazard Evaluation Model was 0.65 ± 0.21. The differences between the RRs obtained from the three models were statistically significant (*F* = 17.598, *P* < 0.001). Compared with the Grading Model, the RRs of the Classification Model and the Occupational Disease Hazard Evaluation Model were significantly higher. The difference was statistically significant (*P* < 0.001). However, the difference between the RRs of the Classification Model and the Occupational Disease Hazard Evaluation Model was not statistically significant (*P* = 0.466). The magnitudes of the RRs of the three models were in the following order: the Classification Model > the Occupational Disease Hazard Evaluation Model > the Grading Model.

The Grading Model and the Occupational Disease Hazard Evaluation Model are similar in principle, both of which combine the inherent risk level and occupational health management levels of enterprises for comprehensive risk assessment. Since the variance analysis showed a statistically significant difference in the risk levels obtained by the two models, the next step of this study was to explore the reasons for the differences between them. The unadjusted risk indicators and the occupational health management indexes of the models were considered. As shown in Table 10, the analysis of R_O_s (for the Grading Model) or the risk indices (for the Occupational Disease Hazard Evaluation Model) of the occupational health management level revealed that the risk indices of the Occupational Disease Hazard Evaluation Model were significantly higher than the R_O_s of the Grading Model, and the difference was statistically significant (*P* = 0.034). The evaluation results of the two risk assessment models on the occupational health management levels of 10 electronic enterprises are shown in Table 11. The differences between the two risk models were not statistically significant (*P* = 0.856). Therefore, the difference between the assessment results of the two models may be due to the inconsistency in the calculation of the inherent risk level of the enterprise.

**Table 10:** Comparison of R_o_s (the grading model) and risk indices (the occupational disease hazards evaluation model).

**Model**	**R**_**o**_ **s or risk indices of different enterprises**										***Z*-value**	***P*-value**
	**A**	**B**	**C**	**D**	**E**	**F**	**G**	**H**	**I**	**J**		
The grading model	0.4	0.4	0.4	0.6	0.4	0.4	0.6	0.4	0.4	0.4	−2.121	0.034
The occupational disease hazard evaluation model	0.8	0.6	0.4	0.6	0.4	0.4	0.6	0.6	0.6	0.6		

R_o_s and Risk Indices are normalized similarly to the risk ratios for comparison purposes.

**Table 11:** Two risk assessment models to evaluate the level of occupational health management standardization in 10 electronic enterprises.

**Model**	**Enterprises**										***Z*-value**	***P*-value**
	**A**	**B**	**C**	**D**	**E**	**F**	**G**	**H**	**I**	**J**		
The grading model	1.00	0.50	0.75	0.50	0.25	0.50	0.50	1.00	0.50	0.50	−0.181	0.856
The occupational disease hazard evaluation model	1.00	0.60	0.60	0.40	0.20	0.40	0.60	0.80	0.80	0.80		

### Accuracy validation of model results

In this study, the average risk level of all of the risk factors present in all positions was used for accuracy verification, and the results of the three models were evaluated for the total risk of the enterprise. Figure 1 shows that the average risk level of all of the risk factors was 0.38 ± 0.18. Comparing the RRs of the three models with the average risk level of all of the risk factors, the results showed that the Classification Model and the Occupational Disease Hazard Evaluation Model did not agree with the average risk level of all of the risk factors, and the difference was statistically significant (*P* < 0.001). On the contrary, the results of the Grading Model did not differ in any way from the average risk level of all of the risk factors (*P* = 0.505), which indicated that the results of the Grading Model more accurately reflected the actual risk of the enterprise.

## Discussion

With the rapid development of the economy, the electronics industry is employing more and more workers, and the occupational health problems of these workers are becoming increasingly prominent (28). Previous studies on the electronics industry have shown that the occupational disease hazards in the electronics industry are mainly organic solvents and noise, and some new occupational disease hazards such as hexane also need to be assessed due to the continuous updating of process technology (29).

Liver damage evidenced by the elevation of alanine aminotransferase and oxidative stress markers has been observed in patients exposed to organic solvents. In addition, chronic or high exposure to organic solvents may be associated with reduced female fertility (30) and hearing organ damage in workers (31). Noise can have direct and cumulative adverse effects that impair health and degrade residential, social, working, and learning environments with corresponding natural (economic) and intangible (welfare) losses (32). Regarding the direct effects, exposure to intense sound or noise may result in a purely temporary threshold shift or cause a residual permanent threshold shifts and alterations in the growth functions of auditory nerve output (33). Regarding the cumulative adverse effects, large epidemiological studies on community noise have reported its association with breast cancer, stroke, type 2 diabetes, and obesity (34). Simultaneous exposure to noise and a mixture of organic solvents may have a secondary effect on the risk of hypertension (35) and additional damage to the auditory organs (36). In this study, the on-site survey of 10 Shenzhen-based electronic companies found that their main occupational hazards were organic solvents and noise. Six companies exceeded the noise standard with an exceedance rate of 60%, indicating that the hazard of noise in the electronics industry was severe and needed to be given high priority, consistent with previous studies (3). Meanwhile, the on-site survey showed that the numbers of workers in the selected enterprises ranged from 300 to 1,900 or more, with workers working up to 11 h, suggesting that the electronics industry was dominated by labor-intensive enterprises, resulting in a high potential risk of occupation-related diseases.

Engineering protections are the primary occupational disease prevention and control measures that can fundamentally control and eliminate the possible occupational hazards in the workplace. Their functions are to prevent workers from being exposed to occupational disease hazards as much as possible or to control the levels of harmful factors in the workplace within the permissible ranges of occupational health standards (37). Besides, personal protective equipment is an important type of protection for workers, and the correct selection and wearing of personal protective equipment is a prerequisite for ensuring the health and safety of workers (38). The on-site survey showed that only 2 out of 10 enterprises complied with the regulations of Chinese occupational health in terms of engineering protection and personal protective equipment, with a compliance rate of only 20%, which was inconsistent with the findings of previous studies on the electronics industry (39). The above results indicate that the electronics industry has poor control of hazard factors. Enterprises should be equipped with self-contained engineering protection facilities and personal protective equipment. Meanwhile, government occupational health supervision departments should strengthen their health supervision.

The application of risk assessment in the field of occupational health is relatively mature, and traditional occupational health risk assessment models—such as the Singapore model, the US EPA quantitative risk assessment model, the ICMM model, the Romanian risk assessment model, and the COSHH essential model—are more accurate in identifying risks of job positions and valuable for helping enterprises quickly implement effective control measures. However, the number of electronics enterprises in China is large, and it does not seem feasible for the supervisory department to urge enterprises to rectify the situation based on job risks. Therefore, it is more effective for regulators to improve efficiency by implementing risk assessment based on the comprehensive risk level of individual enterprises and adjusting the supervision of enterprises with different risk levels accordingly.

The correlation analysis of the three risk assessment models showed that there was no correlation between these three models (*P* > 0.05). Comparing the RRs of the three models, it was found that the Classification Model and the Occupational Disease Hazard Evaluation Model had significantly higher RRs than the Grading Model (*P* < 0.001), which depended on the principle of the model itself. The three comprehensive risk assessment models used in this study have their own advantages and disadvantages due to their different modeling principles. The Classification Model is a qualitative model that classifies the comprehensive risk of an enterprise by the industry classification as well as the types and levels of hazards faced by the enterprises. Its advantage is that it can quickly determine the comprehensive risk of an enterprises, and it is easy for non-specialists to use. Moreover, the Classification Model is sensitive to the identification of severely hazardous occupational hazards (e.g., 1,2-dichloroethane and benzene) and other highly pathogenic or toxic substances, so a higher risk rating may be derived if the above chemical hazards are present in the workplace. This suggests that the results of the Classification Model can work well in preventing workers from developing occupational diseases. However, the assessment results obtained from the Classification Model are crude and do not integrate the actual exposure data of the positions. Both the Grading Model and the Occupational Disease Hazard Evaluation Model are quantitative models that combine the levels of risk of occupational hazards with the occupational management level of the enterprise. The Grading Model combines the principles of various traditional occupational health risk assessment models. Its greatest advantage is that the specific exposure level of the position is fully considered before quantitative calculation, and then the occupational health management level of the enterprise is weighted, so the result may better reflect the actual risk level of the enterprise. However, it is worth noting that its complicated assessment formula could limit its use and promotion. The Occupational Disease Hazard Evaluation Model considers the health effects, likelihood (exposure time and intensity), and severity (health effects) of hazards, as well as the number of people exposed and protective measures, and the enterprise's occupational health management. The model avoids a complicated calculation process, reduces the subjectivity of the assessment to a certain extent, and reflects the current situation of the enterprise as comprehensively as possible. The Grading Model and the Occupational Disease Hazard Evaluation Model are similar, but yield very different risk assessment results (*P* < 0.05). The risk level R_O_ (the Grading Model) and risk index (the Occupational Disease Hazard Evaluation Model) of the two models before the adjustment of the occupational health management level were analyzed, and it was found that assessment result of the Occupational Disease Hazard Evaluation Model was higher than the Grading Model (*P* < 0.05), but there was no difference in the occupational health management level derived from the two risk assessment models (*P* > 0.05). This suggests that the reason for the large difference in the assessment results of the two models may be due to the difference in R_O_ or risk index. In addition, the large difference in risk assessment results between the Grading Model and the Occupational Disease Hazard Evaluation Model could also be due to the difference in the adjustment matrices of occupational health management level. From the matrices of the two models, it can be seen that the Grading Model is more conservative than the Occupational Disease Hazard Evaluation Model, which is reflected by the fact that the Grading Model is less influenced by the occupational health management status of the enterprise when the *R*_*O*_ or risk index is at a low to medium level, and, thus, obtains a lower risk level.

It was found that workers in the in-service group in the electronics industry had significantly higher rates of abnormal blood pressure than those in the pre-employment group (40). Meanwhile, a study analyzed the occupational health results of a street in the electronics industry and found that the current health status of workers in the electronics industry was not optimistic, with a 50% abnormal detection rate (28). In Jiangsu Province, 166 cases of occupational poisoning (including 157 cases of chronic occupational poisoning) occurred in the electronics industry, accounting for 17.2% of occupational poisoning cases in the province (41). Data on the distribution of occupational diseases in the Baoan district of Shenzhen from 2000 to 2011 showed that the composition ratio of the electronics industry (36.8%) was much higher than that of other industries (42). In this study, the average risk level of all of the hazard factors of all positions was analyzed, and the results showed that the average RR was 0.38 ± 0.18, indicating that the risks of the 10 electronics enterprises was at a medium level, which was basically consistent with the results of the above studies. The RRs of the three models were compared with the average risk level of all of the hazard factors of the positions, and there was no difference between the RRs of the Grading Model and the average risk level (*P* > 0.05), which indicated that the Grading Model better reflected the actual risk levels of the electronics enterprises, and the assessment results of the total risk of the enterprises were more scientific and accurate. The other two models may have overestimated the overall risk level of enterprises due to different principles.

## Conclusions

The hazards of organic solvents and noise in the electronics industry deserve great attention, and the occupational health management of enterprises also needs to be improved. The Classification Model and the Occupational Disease Hazard Evaluation Model may overestimate the risk level of electronics enterprises, whereas the results of the Grading Model are more in line with the actual risk of enterprises.

## Data availability statement

The original contributions presented in the study are included in the article/supplementary material, further inquiries can be directed to the corresponding author.

## Ethics statement

Ethical review and approval was not required for the study on human participants in accordance with the local legislation and institutional requirements. Written informed consent from the participants was not required to participate in this study in accordance with the national legislation and the institutional requirements.

## Author contributions

SS, CW, TW, and XL contributed to the data acquisition. SS, QH, XZ, and JZ contributed to data analysis, interpretation, and drafted. SS and QH conceived and designed the study. SS critically reviewed the manuscript for intellectual content and is the guarantor of this work and, as such, had full access to all the data in the study and takes responsibility for the integrity of the data, and the accuracy of the data analysis. All authors reviewed and approved the final version of the manuscript and contributed to the article and approved the submitted version.
